# Supplementary material for: Unraveling Transcriptome Profile, Epigenetic Dynamics, and Morphological Changes in Psoriasis-like Keratinocytes: “Insights into Similarity with Psoriatic Lesional Epidermis”
Source: Cells. 2023 Dec 12;12(24):2825. doi: 10.3390/cells12242825 (PMC10741855; doi:10.3390/cells12242825)
Supplement: Supplementary file 1 [file cells-12-02825-s001.zip › cells-2668059-supplementary.pdf]

Table S1. Real-time RT-PCR TaqMan assay probes

Number	Gene symbol	Assay ID	Catalog Number
**1**	**VIM**	Hs00958111_m1	
**2**	**CLDN1**	Hs00221623_m1	
**3**	**KRT1**	Hs00196158_m1	
**4**	**KRT10**	Hs00166289_m1	
**5**	**KRT15**	Hs00951967_g1	
**6**	**KRT17**	Hs00356958_m1	
**7**	**IL-23A**	Hs00900828_g1	
**8**	**DNMT1**	Hs00945899_m1	4331182
**9**	**DNMT3A**	Hs01027166_m1	
**10**	**DNMT3B**	Hs00171876_m1	
**11**	**TET1**	Hs04189344_g1	
**12**	**TET2**	Hs00325999_m1	
**13**	**TET3**	Hs00896441_m1	
**14**	**HDAC9**	Hs01081558_m1	
**15**	**18s rRNA**	Hs99999901_s1	

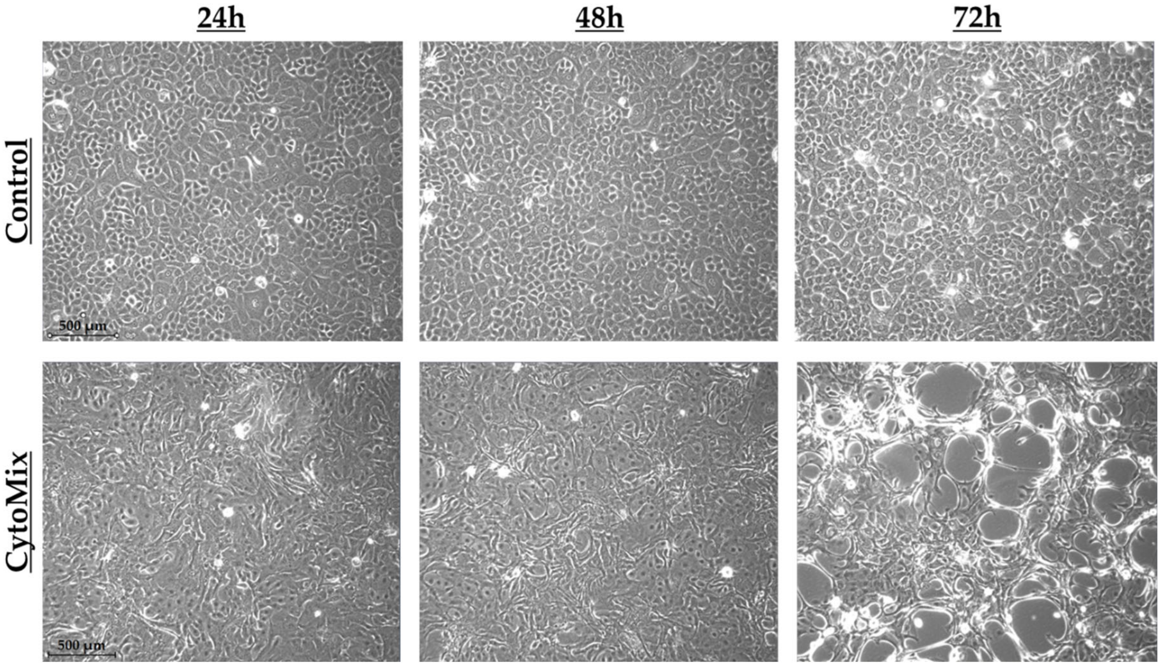

Figure S1.Morphological changes in severely inflamed keratinocytes. . To induce severe inflammation, KCs were treated with 10X CytoMix for 24, 48, and 72 hours. The representative images of three biological replicates are shown under transmitted light microscopy (n=3). Scale bars are 500 µm in Control and CytoMix groups.

Table S2. Enriched pathways in mildly and severely inflamed keratinocytes (KCs) compared to controls across biological process (BP), cellular component (CC), and molecular function (MF) categories.. Abbreviations: BP, biological process, CC, cellular component; MF, molecular function; KCs, keratinocytes.

Group	Go term	p value	Functional group	Number of Genes
Mildly-inflamed KCs	**tissue development**	5.6839E-18	BPs	167
	inflammatory response	3.8299E-13		77
	myeloid leukocyte activation	1.4831E-14		74
	skin development	5.6667E-28		75
	taxis	1.5593E-09		62
	**cytoplasmic vesicle part**	6.0089E-14	CCs	119
	vesicle	3.3224E-18		280
	tertiary granule	1.7841E-05		20
	lysosome	7.3392E-05		49
	plasma membrane	6.4435E-11		302
	**interleukin-1 receptor binding**	1.9859E-06	MFs	7.00
	cytokine receptor binding	1.7496E-07		32.00
	serine-type endopeptidase inhibitor activity	7.0770E-06		15.00
	RAGE receptor binding	2.1181E-05		5.00
	receptor binding	2.6492E-05		100.00

Group	Go term	*p* value	Functional group	Number of Genes
Severely-inflamed KCs	**cellular nitrogen compound metabolic process**	2.8466E-42	BPs	2536.00
	intracellular transport	9.7072E-22		804.00
	gated channel activity	1.2215E-15		77.00
	negative regulation of biological process	3.4488E-12		2048.00
	organonitrogen compound metabolic process	9.8856E-29		2604.00
	**intracellular part**	4.3187E-142	CCs	5190.00
	mitochondrion	1.06014E-28		792.00
	cell periphery	2.72435E-26		1491.00
	organelle membrane	1.62322E-16		1137.00
	extracellular exosome	2.00502E-11		1087.00
	**cation channel activity**	3.7769E-16	MFs	42.00
	G-protein coupled receptor activity	3.1626E-78		67.00
	methyltransferase activity	7.9790E-08		110.00
	serine-type endopeptidase activity	2.8176E-07		57.00
	nucleotide binding	3.9468E-05		909.00

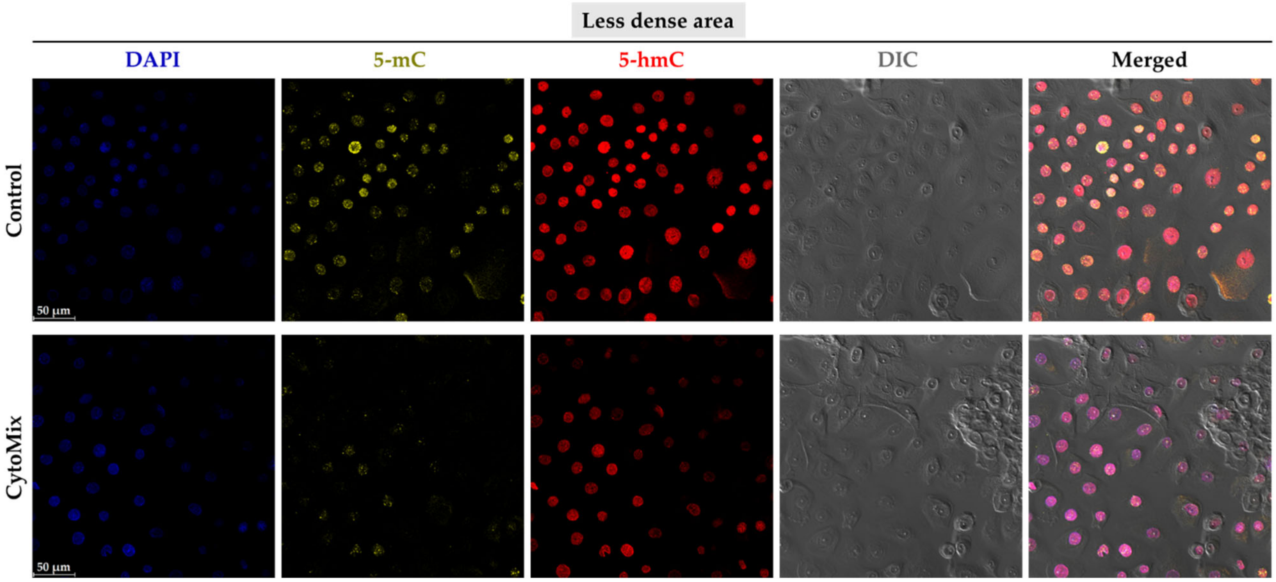

Figure S2. Differential 5-mC and 5-hmC patterns in keratinocytes treated with/without CytoMix in low density areas. The KCs were seeded at a density of 1.0-1.2×10^5 cells/well in 8-well slide chambers and were treated with or without CytoMix for 48 hours to induce mild inflammation. Then, the cells were fixed with 4% PFA and stained for 5-mC (in yellow) and 5-hmC (in red). The nuclei were stained with DAPI (in blue). The confocal microscopy images were taken from control and CytoMix-treated KCs (n=3). Scale bars are 50 µm in both control and CytoMix panels.
